# Characterization and simulation of cDNA microarray spots using a novel mathematical model

**DOI:** 10.1186/1471-2105-8-485

**Published:** 2007-12-20

**Authors:** Hye Young Kim, Seo Eun Lee, Min Jung Kim, Jin Il Han, Bo Kyung Kim, Yong Sung Lee, Young Seek Lee, Jin Hyuk Kim

**Affiliations:** 1 Department of Physiology, College of Medicine, Hanyang University, Seoul, 133-791, Korea; 2 Department of Biochemistry and Molecular Biology, College of Medicine, Hanyang University, Seoul, 133-791, Korea; 3 Division of Molecular & Life Science, College of Science and Technology, Hanyang University, Ansan, 426-791, Korea

## Abstract

**Background:**

The quality of cDNA microarray data is crucial for expanding its application to other research areas, such as the study of gene regulatory networks. Despite the fact that a number of algorithms have been suggested to increase the accuracy of microarray gene expression data, it is necessary to obtain reliable microarray images by improving wet-lab experiments. As the first step of a cDNA microarray experiment, spotting cDNA probes is critical to determining the quality of spot images.

**Results:**

We developed a governing equation of cDNA deposition during evaporation of a drop in the microarray spotting process. The governing equation included four parameters: the surface site density on the support, the extrapolated equilibrium constant for the binding of cDNA molecules with surface sites on glass slides, the macromolecular interaction factor, and the volume constant of a drop of cDNA solution. We simulated cDNA deposition from the single model equation by varying the value of the parameters. The morphology of the resulting cDNA deposit can be classified into three types: a doughnut shape, a peak shape, and a volcano shape. The spot morphology can be changed into a flat shape by varying the experimental conditions while considering the parameters of the governing equation of cDNA deposition. The four parameters were estimated by fitting the governing equation to the real microarray images. With the results of the simulation and the parameter estimation, the phenomenon of the formation of cDNA deposits in each type was investigated.

**Conclusion:**

This study explains how various spot shapes can exist and suggests which parameters are to be adjusted for obtaining a good spot. This system is able to explore the cDNA microarray spotting process in a predictable, manageable and descriptive manner. We hope it can provide a way to predict the incidents that can occur during a real cDNA microarray experiment, and produce useful data for several research applications involving cDNA microarrays.

## Background

With the advance in technology for simultaneous acquisition of information on a number of genes' expression, the research area of bioinformatics is expanding into the reconstruction of a system composed of such genes. Within a decade, the technology of cDNA microarray experiments has been extensively developed, and its usage has exponentially increased. To improve the accuracy of microarray data, a number of techniques in post-processing microarray data were suggested from image analysis to statistical data processing. However, in spite of those efforts, the increase in accuracy of post-processing has a limit. After all, the improvement in wet-lab experiments, such as ensuring the conditions for sound spotting of cDNA probes, is necessary to obtain reliable microarray data. Most cDNA microarray images contain spots in various shapes, including doughnut-shaped spots, which consist of pixels of high intensity at the perimeter and those of low intensity in the central area. Such patterns of spot images are primarily due to the non-uniform distribution of cDNA molecules while the cDNA solution dries out during the microarray printing process.

**Figure 1:**
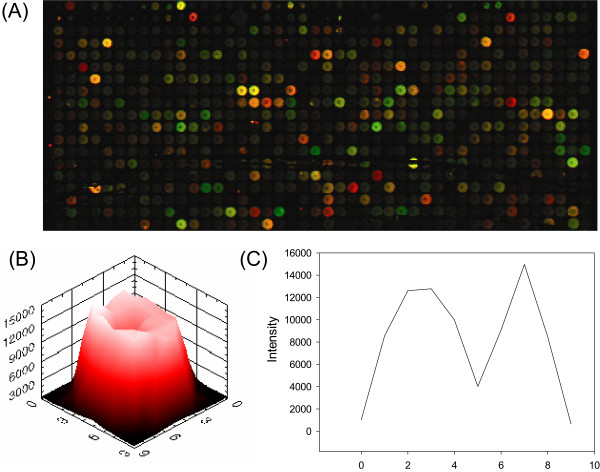
**Various spot shapes in real microarray images**. (A) Real microarray images contain spots in various shapes [38] [40]. (B) The 3D image of spots in the real microarray images were reconstructed by plotting image intensity on the z-axis (C) The radial profiles of the 3D images were obtained where the section passed through the centre.

Spot morphology influences the measurement of gene expression levels. In particular, doughnut-shaped spots among them can often cause errors in measuring the signals. The inner hole can be either a blank or an area composed of pixels of lower signal than those in the perimeter (Figure 1). When calculating the measure of a spot signal, there is a large difference between the inclusion and exclusion of the hole in the spot area. If the hole is a complete blank, the area of the hole should be excluded from the spot area when the spot signal is measured. Otherwise, it should not be excluded. To overcome this, several techniques have been developed for detecting the effective area of a spot [1,2]. They basically require some thresholds or parameters that are critical in determining the effective area. In a microarray image, each of the thousands of spots has a unique morphology and range of pixel intensity. Therefore, it is not proper to use a single method to calculate gene expression levels from the spots in various shapes and characteristics.

Since microarray became widely used for obtaining high-throughput gene expression data, several methods for simulating the microarray experiment have been suggested [3-7]. Simulation is useful for designing and testing both the experiment and the analysis in real fields of study. This makes it possible to predict how the experiment will proceed or how well the analysis will work, before it is realized. In addition, it also provides synthetic data for analysis, when the realization is impossible due to a technical problem. Previous methods focused on mimicking images generated by real cDNA microarray experiments, and their parameters for determining the features of spots were based on certain probabilistic models. Wierling *et al.*[4] classified spots into three categories: convex, crater-like, and cylindrical spots. Balagurunathan *et al.*[3] simulated spots from simple circles and modified them by resizing radii, punching holes in the centre of spots, reshaping spots by removing chords, and enhancing intensities at the edge of spots. However, if it were not based on the investigation of physical and chemical nature, it would fail to correctly simulate and might confuse the experimenters with the results.

In this respect, it is important to study the formation of the variously shaped spots. The phenomenon of deposition of solute drop on the free surface has been studied [8-14]. Deegan et al. explained the formation of a ring-shaped stain when a coffee drop evaporates as a result of "pinning" of the contact line. Heim *et al.*[15] noticed the importance of study of the deposition of DNA unspecifically bound on the microarray slide. However, microarray experiments are performed on slides specially manufactured for the purpose of increasing the sensitivity and the specificity of the results. A capping step to prevent non-specific adsorption on the support during hybridization and to decrease the background noise is often performed. To minimize the loss of cDNA by hybridization or washing out, the surface of a glass slide is usually coated with substrates [16]. Therefore, it is necessary to study the characteristics of cDNA deposition by close investigation focused on the microarray experiment.

In this study, we generate a mathematical model of cDNA deposition during the microarray spotting process using the contact printing technology by bringing parameters from cDNA microarray experiments. This study analyzes cDNA microarray spot formation and elucidates parameters which affect the spot morphology. The study of the origin of various patterns in spot morphology suggests how to manufacture good microarray spots.

## Results

### cDNA deposit model generation

We consider a drop of cDNA solution on a glass slide coated with a chemical layer bearing ended function able to bind strongly with cDNA. While the cDNA solution is spotted on the slide, the strong adhesive forces of the chemical layer pull a drop of cDNA solution reserved in the channel of the spotting pin, and the cDNA molecules bind to the surface sites of the chemical layer so as to be immobilized. While the drop is dehydrated, the concentration of cDNA changes in two ways simultaneously: 1) it increases due to the evaporation of water at the surface of the drop; 2) it also decreases due to the deposition of cDNA molecules on the surface of the glass slide dried out from the rim of the drop. Because water evaporates at an equal rate across the surface of the drop, the rate of change of the volume *V *is proportional to the surface area *S*, such that

(1) $$\frac{dV}{dt}=-K_{E}S$$

where *K*_*E *_is the evaporation constant reflecting the conditions of the experiment, such as temperature, humidity, and atmospheric pressure, etc.

When a drop of cDNA solution is spotted on a glass slide, it has a hemispheric-like shape on the condition of the surface tension of the drop and the hydrophobicity of the original surface of the slide. If we consider the drop as a spherical cap, the volume and the surface area are proportional to the cube and the square of the contact radius *r*, such that *V*(*r*) = *K*_*V*_*r*^3 ^and *S*(*r*) = *K*_*S*_*r*^2^. *K*_*V *_and *K*_*S *_are the volume and the surface constants, respectively, such that

(2) $$\begin{array}{l} K_{V}=\frac{1}{3}\pi \csc^{3}\theta {\left(1-\cos\theta \right)}^{2}\left(2+\cos\theta \right)\\ K_{S}=2\pi \csc^{2}\theta \left(1-\cos\theta \right) \end{array}$$

where *θ *is the contact angle of drop on the slide. Because the drop undergoes morphological transition during evaporation, the contact angle is a function of time *t*, *θ *= *θ *(*t*), and the rate of change in volume is

(3) $$\frac{dV}{dt}=3K_{V}r^{2}\frac{dr}{dt}+r^{3}\frac{dK_{V}}{dt}.$$

Several studies have investigated the morphological transition of a drop during its evaporation [17-20]. At the beginning of evaporation, the contact line is pinned, i.e., while the contact radius remains constant, the drop diminishes in volume as the contact angle and the drop height decrease (State I). When the contact angle and the drop height decrease to a certain level, the contact line is depinned, i.e., the contact radius starts to retract with the decrease of drop height and the contact angle becomes constant (State II).

At state I, cDNA molecules bind with the surface sites in the whole contact area of the drop in the chemical equilibrium state, as follows:

(4) $$\begin{array}{c} \underset{\rho }{cDNA}+\underset{x_{0}-x}{SurfaceSite}\overset{k_{+}}{\underset{k_{-}}{⇄}}\underset{x}{cDNA\cdot SurfaceSite}\\ \begin{array}{ccc} \rho =\frac{x}{Ke^{-ax}\left(x_{0}-x\right)} & for & K=k_{+}/k_{-} \end{array} \end{array}$$

where *x*_0 _is the surface site density on the support and *a *is the interaction factor for the non-ideal adsorption behaviour of macromolecules [21]. If *a *= 0, the equation is the same as the Langmuir adsorption equation, and otherwise, it describes the non-ideal adsorption behaviours, such as repulsion (*a *> 0) and attraction (*a *< 0) between molecules [22,23]. Therefore, the equilibrium constant for the binding reaction is *Ke*^-*ax*^, where *K *is the extrapolated equilibrium constant. The concentration of cDNA, *ρ*, rises during evaporation without reduction of the radius of the drop, and, therefore, the quantity of cDNA molecules, *x*, which bind with the surface sites in a unit area increases nonlinearly.

At state II, as the contact line moves back, the cDNA molecules start to deposit at the contact line. The recession of the contact line prevents cDNA molecules from dissolving back into the drop. Because the contact angle becomes constant, *K*_*V *_and *K*_*S *_do not vary with time, and, therefore, the second term on the right-hand side of the equation (3) can be omitted. Then the rate of change in the contact radius can be also constant, as in

(5) $$\frac{dr}{dt}=-\frac{1}{3}K_{E}K_{S}/K_{V}=-\kappa .$$

Let *ρ*(*t*) represent the concentration of cDNA at time *t *when the contact radius becomes *r*(*t*). If we assume that the density of the surface sites is constant in the contact area of a given drop, then

(6) $$\begin{array}{c} \rho (t)=\frac{Initialquantity\;of\;cDNA-DepositedcDNA}{Initialvolumeofdrop-Evaporated\;water}\\ =\frac{\rho (0)V_{0}-\int_{0}^{t}2\pi r\left(\tau \right)x\left(\tau \right)\kappa d\tau }{V_{0}-\int_{0}^{t}K_{E}Sd\tau } \end{array}$$

where *ρ*(0)is the cDNA concentration at the moment beginning deposition, and *V*_0 _is the initial volume of the drop. Then, equation (6) can be simplified under the assumption that the contact angle is constant after the beginning of cDNA deposition, such as

$$\frac{d}{dt}\rho \left(t\right)=\frac{3\kappa }{r_{0}-\kappa t}\rho \left(t\right)-\frac{2\pi \kappa }{K_{V}{\left(r_{0}-\kappa t\right)}^{2}}x\left(t\right)$$

where *r*_0 _= *r*(0). From the temporal derivative of the concentration of the drop, we can derive its radial derivative, as in

(7) $$\frac{d\rho }{dr}=-3\frac{\rho }{r}+\frac{2\pi }{K_{V}}\frac{x}{r^{2}}.$$

The radial derivative of the deposit can be obtained from equation (4) and (7), as follows:

(8) $$\frac{dx}{dr}=\frac{x^{2}}{x_{0}\rho Ke^{-ax}+ax^{2}}\left(-\frac{3}{r}+\frac{2\pi x}{K_{V}r^{2}\rho }\right).$$

The solution of equation (8) is the density of cDNA deposit at the distance *r *from the centre, which can be solved by an ordinary differential equation solver with the four parameters, *x*_0_, *K*, *a*, and *K*_*V*_.

### Spot simulation

**Figure 2:**
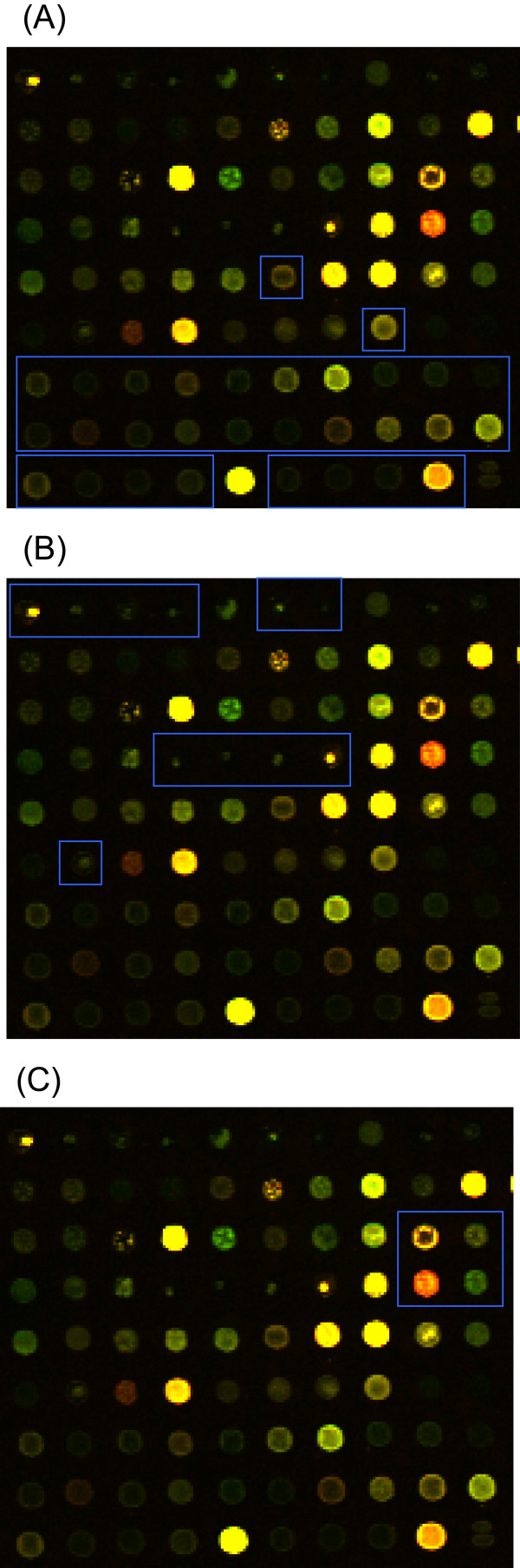
**Three types of spot morphology in a real microarray image**. (A) The doughnut-shaped spot. (B) The peak-shaped spot. (C) The volcano-shaped spot.

**Figure 3:**
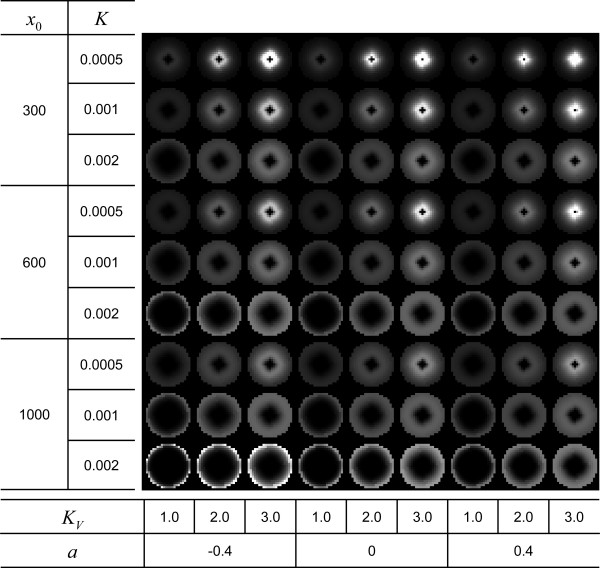
**Simulated cDNA deposit in various shapes**. By varying parameters *x*_0_, *K*, *a*, and *K*_*V *_with three different values, we obtained a total of 81 different shapes of cDNA deposit. The values of parameters used for simulating each shape of cDNA deposit are listed in the tables on the left and the bottom of the image.

From a real microarray image [24], we identified the three types of spots (Figure 2). Using the radial cDNA deposit profile obtained from equation (8) with varying parameters, *x*_0_, *K*, *a*, and *K*_*V*_, we obtained several types of radial cDNA deposit profiles (Figure 3).

### Doughnut-shaped spot

**Figure 4:**
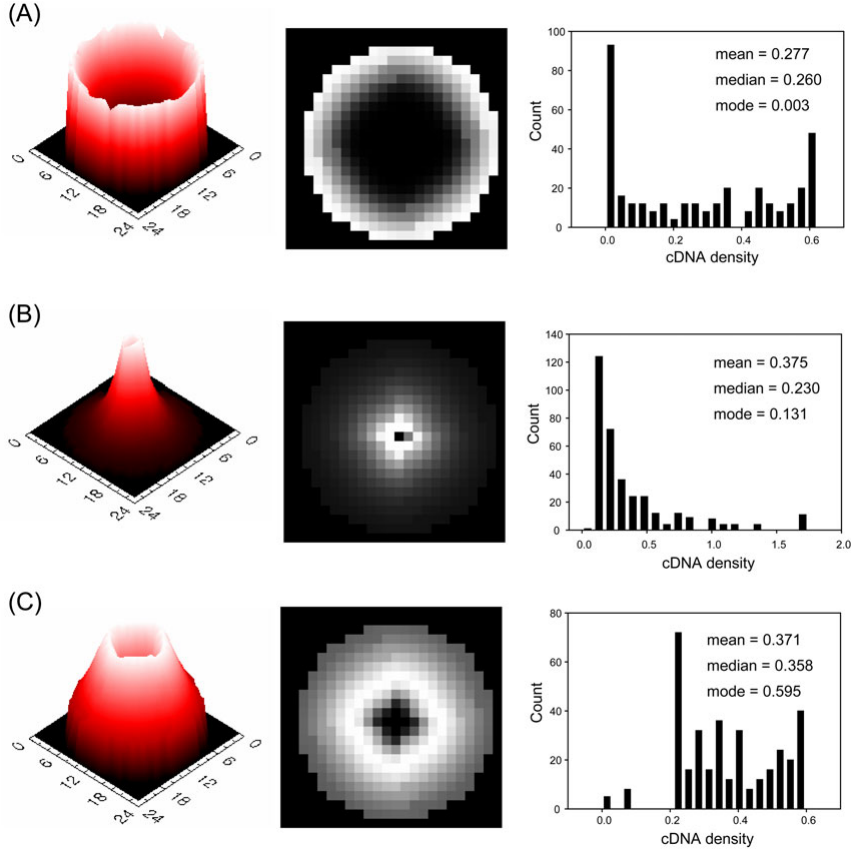
**Three types of morphology of simulated cDNA deposit**. (A) The doughnut-shaped spot was generated with *x*_0 _= 1000, *K *= 0.002, *a *= 0.4, and *K*_*V *_= 2.0. (B) The peak-shaped spot was generated with *x*_0 _= 300, *K *= 0.001, *a *= 0.4, and *K*_*V *_= 3.0. (C) The volcano-shaped spot was generated with *x*_0 _= 1000, *K *= 0.0005, *a *= -0.4, *K*_*V *_= 3.0, and. Here, mean, median and mode are the arithmetic mean, the value of the middle term ranked in increasing order, and the value that occurs with the highest frequency, respectively.

**Figure 5:**
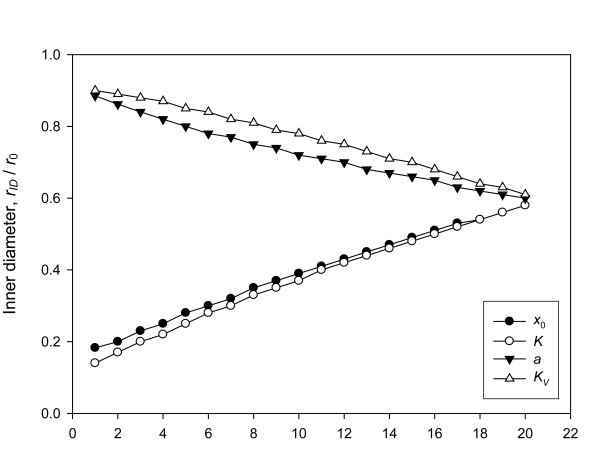
**Changes in inner diameter of doughnut-shaped spot**. The default values of the parameters were set as *x*_0 _= 1000, *K *= 0.002, *a *= 0.4, and *K*_*V *_= 3. By decreasing the value of each parameter with 20 steps and fixing the other four parameters with the default values, we obtained 20 variously shaped spots and measured the inner diameters of the spots. The decrements of *x*_0_, *K*, *a*, and *K*_*V *_were set as 35, 7.5E-5, 0.04, and 0.1, respectively. The inner diameter was measured at the height of half of the maximum intensity.

The doughnut-shaped spot was defined as the spot having a thin rim of high density of deposited cDNA and a large hole of very low density, approximated to zero (Figure 4a, left and middle). The diameters of the inner hole of the doughnut-shaped spots were measured by varying the parameters. Histograms of the density of deposited cDNA were bimodal, which had a peak at zero (Figure 4a, right). If the inner hole is included in measuring the signal, then there will be a discrepancy between the measures and the true value. To verify which parameter can influence the morphology of the doughnut-shaped spot, we changed each parameter by 20 steps while the others were fixed. The inner hole was decreased by increasing *a*, and *K*_*V *_and decreasing *x*_0 _and *K *(Figure 5).

### Peak-shaped spot

The peak-shaped spot was defined as a spot having the highest density at the central region and a declining density at the region farther from the centre (Figure 4b, left and middle). Because the peak-shaped spot can have an obscure boundary with the background, the edge of the spot can often be determined by the threshold. The histogram of the density of deposited cDNA was skewed to the right (Figure 4b, right). Therefore, the mean density of deposited cDNA is lower than the median value. Furthermore, the measure can vary with how large an area of spot is included.

### Volcano-shaped spot

We defined the peak-shaped spot having a small hole in its peak as the volcano-shaped spot because its 3D image resembles a volcano (Figure 4c, left and middle). The volcanic-shaped spot has characteristics of both the doughnut-shaped spot and the peak-shaped spot (Figure 4c, right).

### Simulated cDNA deposit

**Figure 6:**
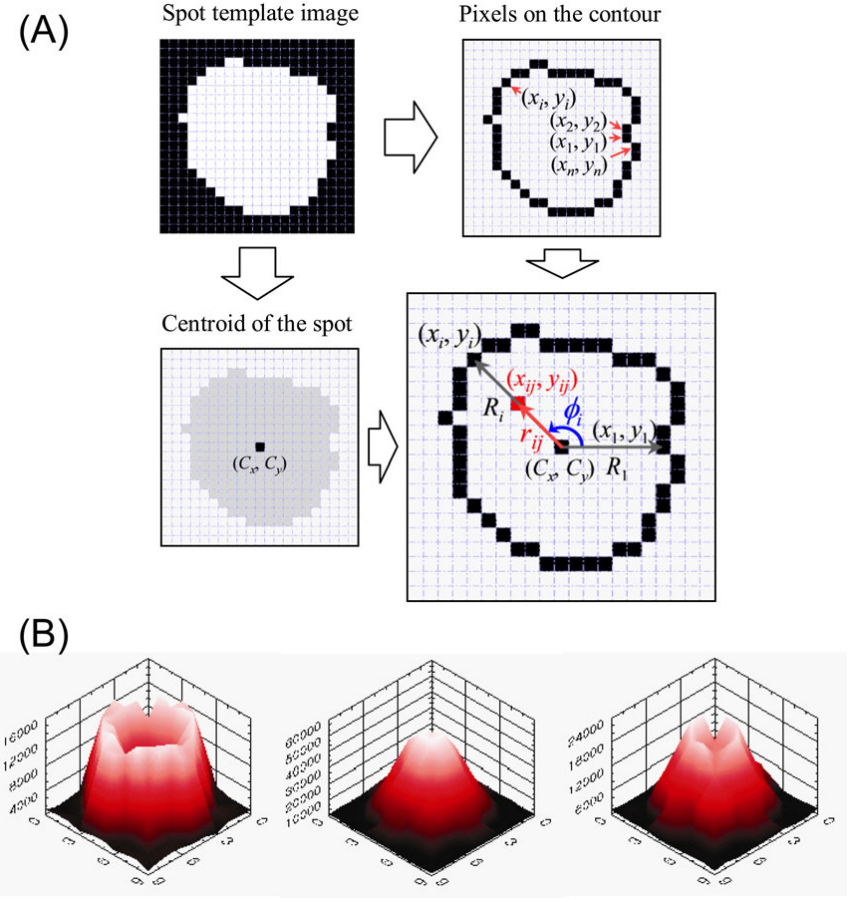
**Simulated cDNA deposit**. From the profile of cDNA deposits and the spot template images, the cDNA deposit was simulated. (A) We set (*x*_*i*_, *y*_*i*_), the coordinates of pixels on the edge contour, where *i *= 1, 2,..., *n*; (*C*_*x*_, *C*_*y*_), centre of the spot area; *R*_*i*_, the distance from the centre to the *i*^*th *^pixel on the edge contour; and *r*_*ij*_, the distance from the centre to each pixel *in *the spot area, where *j *= 0, 1,..., *R*_*i*_. (B) The 3D images of the deposited cDNA were visualized.

Using the radial profile of cDNA deposits and the spot template images, we simulated the deposition of cDNA in a virtual spot. In each spot template image, we set coordinates of pixels on the edge contour and centre of the spot area (Figure 6a). Using the distance from the centre to each pixel on the edge contour, we calculated cDNA concentration at the moment when the contact radius becomes *r*, and the quantity of cDNA molecules that bind with the surface sites in a unit area (Figure 6b).

### Parameter estimation from real microarray images

The spot morphology varies with the size of the end of the spotting pin tip, the volume of sample delivered, the surface tension of the drop, the hydrophobicity of the surface of the slide, and so on. It has been proven that the increase in viscosity and contact angle of a micro-drop shrinks the initial size of the drop [25]. Using the data and equation (2), we calculated the volume constants and the contact angles of the common micro-drops at the moment of being spotted on the glass slides, with the data of the spot diameters and the volumes of delivered sample provided by TeleChem International, Inc. [26]. We found that the initial drop in state I has a wide variety in morphology, from a sphere-like shape with a small contact area (*K*_*V *_= 16.38, *θ *= 141.14°) to a flat spherical cap shape with a large contact area (*K*_*V *_= 0.46, *θ *= 32.01°).

We estimated the four parameters from the real microarray spot images. Our model is one of the constrained nonlinear optimization (CNO) problems, so that the optimum values of the parameters of the ordinary differential equation were obtained by sequential quadratic programming (SQP) [27], with the least squares method, as follows:

(9) $$\begin{array}{cc} minimize & m\left(\theta \right)=\sum_{i=1}^{N}{\left(y_{i}-x\left(r_{i},\theta \right)\right)}^{2} \end{array}$$

(10) *subject to eq*.(8)

where *θ *= (*x*_0_, *K*, *a*, *K*_*V*_), *y*_*i *_is the normalized pixel intensity of a real microarray spot at distance *r*_*i *_from the centre of the spot. SQP was performed on the spots of which outer rims are circular using their radial profiles (as shown in Figure 1c). Because the number of pixels in a spot is not enough to fit the model, we inserted 16 dummy data points between every pixel point on the radial profile by using cubic Hermite spline interpolation [28]. From each image, five spots representing each type of spot shape were selected. The results of the study of the microarray scanning process indicated that the range of the concentration of mRNA of thousands of genes in a sample is much wider than the dynamic range of microarray scanners [29]. If the microarray scanner reads the signal out of the linear dynamic range, the radial profile of the spot deposit would be distorted. To focus on the physical phenomenon of the deposition of cDNA molecules, the fitting was performed on a limited number of spot images whose pixel intensities were proper to be within the linear dynamic range. The estimated values of *x*_0 _and *K *of the doughnut-shaped spots were about ten times as high as those of the peak-shaped spots, while *a *and *K*_*V *_of the former were lower than those of the latter (Table 1 and 2). The macromolecular interaction factor, *a*, is usually a positive value because cDNA molecules are usually negatively charged, and so there is a repulsive force between cDNA molecules. However, their electrical characteristics can be changed, depending on the pH of the buffer solution. The estimated parameters of the volcano-shaped spots were highly variable. Because the volcano-shaped spots contained the characteristics of both the doughnut-shaped spot and the peak-shaped spot, they have a wide variety in the morphology. Table 1 and Table 2 show that the value of *x*_0 _is also highly dependent on the slide.

**Table 1:** Estimated values of the four parameters of the three types of spot shapes of Image I *

Spot shape		*x*_0_	*K*	*a*	*K*_*V*_	MSD***
doughnut	1	101.06	5.67E-3	-0.40	1.74	3.01E-2
	2	540.79	6.35E-3	-0.77	0.41	4.58E-3
	3	635.04	7.35E-3	-0.47	0.71	2.71E-2
	4	686.56	5.66E-3	-0.51	0.44	5.24E-3
	5	777.05	2.73E-3	-0.56	0.64	1.69E-2
	mean	548.10	5.55E-3	-0.54	0.79	1.68E-2
	SD**	264.12	1.72E-3	0.14	0.55	1.19E-2

volcano	1 ^†^	599.58	2.76E-3	-0.55	2.45	8.12E-4
	2 ^†^	265.87	1.61E-3	0.70	2.82	8.96E-3
	3 ^†^	401.92	1.59E-3	-0.72	0.60	3.50E-3
	4 ^††^	32.69	5.23E-3	0.14	3.31	4.21E-3
	5 ^††^	27.10	7.30E-3	0.65	5.43	4.57E-4
	mean	265.43	3.70E-3	0.04	2.92	3.59E-3
	SD**	245.59	2.50E-3	0.66	1.74	3.42E-3

peak	1	39.41	2.55E-4	0.04	4.59	2.26E-2
	2	39.87	2.90E-4	0.18	1.12	1.14E-3
	3	37.51	8.42E-4	0.22	2.68	7.56E-3
	4	37.59	8.41E-4	0.22	2.21	1.13E-3
	5	41.39	2.24E-4	0.86	2.45	1.06E-2
	mean	39.15	4.90E-4	0.30	2.61	8.61E-3
	SD**	1.64	3.21E-4	0.32	1.26	8.83E-3

* This image was obtained from Stanford MicroArray Database [38]: Experiment ID, 50150; Experiment name, MMAF array batch on PLL QC-1; Category, Quality Control; Subcategory, Array Batch QC; Slide name, mmaf042.

** Standard deviation.

*** Mean squared deviation, $\text{MSD}=\frac{1}{N}\sum_{i=1}^{N}{\left(y_{i}-x_{i}\right)}^{2}$.

^† ^The spot shape was close to the doughnut-shape.

^†† ^The spot shape was close to the peak-shape.

**Table 2:** Estimated values of the four parameters of the three types of spot shapes of Image II *

Spot shape		*x*_0_	*K*	*a*	*K*_*V*_	MSD***
doughnut	1	138.07	2.86E-3	-0.89	0.21	3.72E-2
	2	144.76	1.73E-3	-0.74	0.30	1.08E-2
	3	198.19	1.68E-3	-0.65	0.48	1.70E-2
	4	294.20	3.01E-3	-0.26	0.36	2.60E-2
	5	367.35	3.88E-3	-0.37	1.10	2.77E-2
	mean	228.51	2.63E-3	-0.58	0.49	2.37E-2
	SD**	99.63	9.31E-4	0.26	0.35	1.02E-2

volcano	1 ^†^	41.02	1.78E-2	-0.14	1.35	2.43E-2
	2 ^†^	433.28	6.11E-2	-0.08	1.68	7.35E-3
	3 ^†^	467.53	2.24E-3	-0.17	2.96	6.77E-3
	4 ^††^	60.19	4.40E-3	-0.18	2.60	1.09E-2
	5 ^††^	62.10	6.17E-3	-0.01	1.59	1.43E-2
	mean	212.82	1.83E-2	-0.12	2.04	1.27E-2
	SD**	217.38	2.46E-2	0.07	0.70	7.14E-3

peak	1	56.92	1.59E-3	0.28	3.51	2.69E-3
	2	69.96	1.68E-4	0.31	3.02	2.36E-3
	3	58.17	5.02E-4	0.38	2.44	1.55E-3
	4	60.20	3.81E-4	0.73	1.75	1.92E-3
	5	52.05	4.66E-4	0.35	2.39	9.64E-4
	mean	59.46	6.22E-4	0.41	2.62	1.90E-3
	SD**	6.59	5.59E-4	0.19	0.67	6.78E-4

* This image was obtained from Stanford MicroArray Database [38]: Experiment ID, 50151; Experiment name, MMAF array batch on PLL QC-2; Category, Quality Control; Subcategory, Array Batch QC; Slide name, mmaf210.

** Standard deviation.

*** Mean squared deviation, $\text{MSD}=\frac{1}{N}\sum_{i=1}^{N}{\left(y_{i}-x_{i}\right)}^{2}$.

^† ^The spot shape was close to the doughnut-shape.

^†† ^The spot shape was close to the peak-shape.

## Discussion

Unlike the ring-shaped stain formed during the evaporation of a coffee drop, cDNA microarray images produce spots of various shapes, even in the same image. A solution to the coffee drop problem can partly explain the doughnut-shaped spot, which is one extreme of spot morphology in cDNA microarray images. Doughnut-shaped spots have frequently appeared in microarray images. In spite of the effort to prevent such spots from being generated, there is still an uneven density of signals in a single spot. In this study, we devised a generalized mathematical model that can manifest a wide spectrum of spot morphology.

The doughnut-shaped spot would be produced by experiment under the condition that a large quantity of cDNA is deposited early on, and so the concentration of the drop solution is quickly lowered. Such a situation can occur when the adsorption reaction is facilitated by a high surface site density, a large equilibrium constant of the binding reaction, and a low repulsion or attraction between cDNA molecules, or when the initial drop has a flat spherical cap shape. Unlike the doughnut-shaped spot, the peak-shaped spot would be produced under the condition that cDNA is not likely deposited early on, and the concentration of the drop solution continually increases due to the accumulated cDNA, and so the quantity of the deposited cDNA can be the maximum at the centre. Such a situation can occur when the adsorption reaction is impeded by a low surface site density, a small equilibrium constant of the binding reaction, and a high repulsion between cDNA molecules, or when the initial drop has a sphere-like shape.

It is known that the composition of buffer solution changes the spot morphology [30-33]. The optimal composition of buffer solution has been investigated by including detergent or betaine to 3 × SSC or 50% dimethyl-sulfoxide (DMSO). It is known that DMSO decreases the surface tension of drop solution during evaporation, while both 3 × SSC and 3 × SSC with betaine increase the surface tension [32]. The decrease of surface tension is accompanied with a decrease in the contact angle and *K*_*V*_. Moreover, DMSO denatures DNA to be bound well with surface sites [34], which means the increases of the equilibrium constant for the binding reaction, *Ke*^-*ax*^. Putting them together, DMSO would decrease *K*_*V *_and *a*, and increase *K*. Figure 3 and 5 show that the doughnut shape is aggravated by increasing *K *and *x*_0 _and decreasing *K*_*V *_and *a*. As a result, DMSO would aggravate the doughnut shape. In addition, there is a study that the length and the sequence arrangement of DNA molecules would influence on the morphological organization of the deposit [35]. The macromolecular interaction factor, *a*, would reflect the length and sequence arrangement of DNA molecules.

The extrapolated equilibrium constant for the binding reaction *K *also reflects the condition of the slide surface. While homemade poly-L-lysine-coated glass slides have been widely used, several commercial microarray slides are now preferred, such as the FMB cDNA slide (Full Moon Biosystems Inc.), ArrayIt SuperAmine and SuperAldehyde slides (TeleChem International, Inc.). It is known that CMT-GAPS™ slides (Corning) makes the spot morphology much more uniform by preventing doughnut shape formation [34]. It can be assumed that the slides are manufactured to have the optimal binding efficiency. The surface site density, *x*_0_, affects the efficiency of cDNA binding. The increase in *x*_0 _facilitates the forward reaction of equation (4) and vice versa.

The uneven density of cDNA can cause errors in measuring the ratio of gene expression. Let *γ*_3 _and *γ*_5 _be the concentration of mRNA of the control (*C*y3) and experiment samples (*Cy*5), respectively. The quantity of hybridized mRNA with cDNA in a unit area is determined in an equilibrium state, such as

(11) $$\begin{array}{c} \underset{\gamma_{3}}{Cy3}+\underset{\gamma_{5}}{Cy5}+\underset{x-x_{3}-x_{5}}{cDNA}\overset{k_{h+}}{\underset{k_{h-}}{⇄}}\underset{x_{3}}{Cy3\cdot cDNA}+\underset{x_{5}}{Cy5\cdot cDNA}\\ \begin{array}{ccc} \frac{\gamma_{5}}{\gamma_{3}}=\frac{1}{K\left(x-x_{3}-x_{5}\right)}{\left(\frac{x_{3}}{\gamma_{3}}\right)}^{2}\frac{x_{5}}{x_{3}} & for & K=k_{h+}/k_{h-} \end{array} \end{array}$$

where *x *is the local density of cDNA, and *x*_3 _and *x*_5 _represent the quantity of hybridized mRNA of both samples in a unit area. In equation (11), the ratio of *x*_5 _to *x*_3 _can be linear to the ratio of mRNA concentration only when the quantity of hybridized mRNA can be approximated to be linear to the concentration of mRNA in the solution. Where the density of cDNA is much lower, such as in the centre of the doughnut-shaped spot, the quantity of the hybridized mRNA is saturated early in this region. Because the concentration of mRNA is uniform in the solution, the quantity of hybridized mRNA cannot be linear to the concentration of mRNA. Then the ratio of the signal fails to reflect the original ratio of mRNA expression. When we assume that *x*, the local density of cDNA, is much larger than (*x*_3 _+ *x*_5_), equation (11) can be simplified, as follows:

(12) $$\frac{\gamma_{5}}{\gamma_{3}}=\frac{1}{K_{h}x}{\left(\frac{x_{3}}{\gamma_{3}}\right)}^{2}\frac{x_{5}}{x_{3}}.$$

We have estimated parameters from real microarray images, but not all spot images were successful. It was practically impossible to fit our model equation to the doughnut-shaped spots, whose centre part has significantly high intensity. Table 1 and Table 2 show the mean squared deviation (MSD) between real and estimated data, as the goodness-of-fit measure. MSDs obtained from the doughnut-shaped spots were relatively larger than those of the other spots. We expect this problem originates from two major reasons: the effect of diffusion when the microarray slide passes through the hybridization process, and nonlinear transformation of spot signals to image intensity when the region out of dynamic range of the microarray scanner was used in converting spot signals. The effect of hybridization on the change in cDNA density is still controversial. Tran *et al.*[36] claimed that DNA spotted on a glass slide diffuses during hybridization. However, this conflicts with the opinion of Pappaert *et al.*[37], who claimed that the doughnut shape occurs not only from the uneven distribution of DNA deposition but also from the hybridization process. We observed a few microarray images that have concentric circles in the spots. Even though we did not include such phenomenon in this study, it is considered a result of the repetition of pinning and depinning of the contact line [10], and should be investigated further.

## Conclusion

We developed a governing equation that can explain the dynamics of cDNA deposition during evaporation of a drop in the microarray spotting process. Experimental conditions were parameterized and included in the governing equation. The parameters determining the spot morphology were brought from the physical and chemical factors. This explains how various spot shapes can exist and suggests which parameters are to be adjusted for obtaining a good spot. This system is able to explore the cDNA microarray spotting process in a predictable, manageable and descriptive manner. We hope it provides a way to predict the incidents that can occur during a real cDNA microarray experiment, and produce useful data for several research applications involving cDNA microarrays.

## Methods

### Mathematical model

Equation (2) was obtained as follows: consider a spherical cap which has the radius of sphere *R*, and cap height *h*. Then the volume *V *and the surface area *S *are

$$\begin{array}{c} V=\frac{1}{3}\pi h^{2}\left(3R-h\right)\\ S=2\pi Rh. \end{array}$$

When we consider the contact radius *r *and the contact angle *θ*, then

*R *= *r *csc*θ*

*h *= *R*(1 - cos*θ*) = *r *csc*θ*(1 - cos*θ*).

Then the volume and the surface area are the functions of the contact radius and the contact angle, such as

$$\begin{array}{c} V=\frac{1}{3}\pi r^{3}\csc^{3}\theta {\left(1-\cos\theta \right)}^{2}\left(2+\cos\theta \right)\\ S=2\pi r^{2}\csc^{2}\theta \left(1-\cos\theta \right). \end{array}$$

Equation (5) was obtained by combining equation (1) and (3), such that

$$r^{3}\frac{dK_{V}}{dt}+3K_{V}r^{2}\frac{dr}{dt}=-K_{E}K_{S}r^{2}.$$

Because the contact angle is constant at state II, *K*_*V *_does not vary with time, and therefore, the first term on the left-hand side of the equation above can be omitted, as follows:

$$3K_{V}r^{2}\frac{dr}{dt}=-K_{E}K_{S}r^{2}.$$

### Tools for analysis and visualization

The ordinary differential equation, equation (8) was solved by applying the backward differentiation formula (BDF) method. cDNA deposits were simulated with a radius of 100 *μ*m, which were converted to images at a resolution of 10 *μ*m/pixel using LabVIEW and NI-Vision 8.2 (National Instrument, Inc.). The templates of spots were obtained by using the edge detection technique [1]. BDF, CNO, and cubic Hermite spline interpolation were implemented with tools in LabVIEW.

### Data sources

All of the real microarray images were obtained from Stanford MicroArray Database [38]. The data of the spot diameters and the volume of the initial drop of cDNA solution were obtained from TeleChem International, Inc. [39].

## Authors' contributions

HYK, SEL and JHK are responsible for writing the manuscript. MJK, JIH and BKK performed simulation and visualization. YSL^2 ^and YSL^3 ^were involved in discussions and provided experiment data. All authors participated in the study and approved the final manuscript.
